# Comparative study of two protocols for quantitative image-analysis of serotonin transporter clustering in lymphocytes, a putative biomarker of therapeutic efficacy in major depression

**DOI:** 10.1186/s40364-017-0107-6

**Published:** 2017-09-22

**Authors:** Raquel Romay-Tallon, Tania Rivera-Baltanas, Josh Allen, Jose M. Olivares, Lisa E. Kalynchuk, Hector J. Caruncho

**Affiliations:** 1 College of Pharmacy and Nutrition, University of Saskatchewan, 107 Wiggins Road, Saskatoon, SK S7N 5E5 Canada; 2 Neuroscience Cluster, University of Saskatchewan, Saskatoon, Canada; 3 Department of Psychiatry, Alvaro Cunqueiro Hospital, Vigo, Galicia Spain; 4 College of Medicine, University of Saskatchewan, Saskatoon, Canada

**Keywords:** Biomarkers, Depression, Blood smears, Serotonin transporter, Lymphocytes, Membrane protein clustering

## Abstract

**Background:**

The pattern of serotonin transporter clustering on the plasma membrane of lymphocytes extracted from human whole blood samples has been identified as a putative biomarker of therapeutic efficacy in major depression. Here we evaluated the possibility of performing a similar analysis using blood smears obtained from rats, and from control human subjects and depression patients. We hypothesized that we could optimize a protocol to make the analysis of serotonin protein clustering in blood smears comparable to the analysis of serotonin protein clustering using isolated lymphocytes.

**Results:**

Our data indicate that blood smears require a longer fixation time and longer times of incubation with primary and secondary antibodies. In addition, one needs to optimize the image analysis settings for the analysis of smears. When these steps are followed, the quantitative analysis of both the number and size of serotonin transporter clusters on the plasma membrane of lymphocytes is similar using both blood smears and isolated lymphocytes.

**Conclusions:**

The development of this novel protocol will greatly facilitate the collection of appropriate samples by eliminating the necessity and cost of specialized personnel for drawing blood samples, and by being a less invasive procedure. Therefore, this protocol will help us advance the validation of membrane protein clustering in lymphocytes as a biomarker of therapeutic efficacy in major depression, and bring it closer to its clinical application.

## Background

In a seminal and classical report, [1] described the plasma membrane as a lipid bilayer (which is mostly composed of phospholipids and sterols) with embedded proteins that can span the membrane (transmembrane proteins) or attach to it peripherally (peripheral proteins). The structure of the membrane was described as a “fluid mosaic”, which allowed a certain degree of mobility for both lipids and proteins. The presence of tight junctions, the link of membrane proteins to the cytoskeleton, and the clustering of proteins within lipid rafts define specific membrane domains and allow for protein-protein interactions that influence their physiological roles [2]. Among the proteins that tend to be clustered within specific membrane domains are numerous neurotransmitter transporters and receptors that locate in pre- and/or post-synaptic sites, and that in many cases (e.g. transporters and G-Protein-coupled-receptors) accumulate in lipid rafts [3]. The partitioning of these molecules into different membrane compartments has important consequences for their physiological actions, to the extent that alterations in membrane protein clustering may be a marker of cellular physiology/pathophysiology.

In a series of recent reports, we have proposed that alterations in membrane protein clustering in peripheral lymphocytes may represent a novel biomarker of depression: We observed that the pattern of clustering of both the serotonin transporter (SERT) and serotonin 2A receptor on the plasma membrane of lymphocytes from naïve depression patients identified two subpopulations of patients that showed a different therapeutic response after 8 weeks of conventional antidepressant treatment [4–7]. We concluded that analysis of membrane protein clustering in lymphocytes could predict which patients are more likely to respond to antidepressant treatment. This issue is quite important as more than 1/3 of patients fail to show a proper response to antidepressant medication, and this response can only be determined after several weeks of antidepressant use (see [8]). If the pattern of protein clustering in peripheral lymphocytes can accurately predict therapeutic responsivity in patients with depression, this would offer a significant advancement over the hit and miss approach often used in the clinic to map out a course of treatment for individual patients.

One of the challenges associated with the collection and proper preservation of blood samples from the depression patients used in our previous experiments was that both patients and clinical personnel found the drawing of blood samples and isolation of lymphocytes to be both cumbersome and expensive. For research purposes, the traditional drawing of blood samples requires the presence of a trained professional, which increases the cost and complexity of the research project. And for the eventual clinical application of this approach for evaluating potential treatment options for patients, one needs to have a simple technique that can be implemented in any medical clinic. Therefore, we developed a new protocol for the study of SERT clustering that can be done using blood smears (from human and rodents) rather than whole blood samples. In this manuscript, we describe the blood smear protocol in detail and compare the results of SERT labelling in blood smears with those from the analysis of isolated lymphocytes in laboratory rats, and in control human subjects and depression patients.

## Methods

### Immunocytochemistry of SERT in whole blood isolated lymphocytes

The analysis was carried out on a total of 10 healthy subjects and 5 naïve depression patients. All the procedures were carried out in compliance with the Code of Ethics of the World Medical Association (Declaration of Helsinki) and approved by the Alvaro Cunqueiro Hospital Ethics Committee (Spain), which includes a consent form signed up for all participants. The blood was drawn by a trained nurse with the use of a 10 ml BD-Vacutainer ® glass whole-blood tube containing 1.5 ml ADC solution [tri-sodium citrate (22 g/l), citric acid (8 g/l), and dextrose (24.5 g/l)]. Samples were diluted 1:1 in phosphate buffered saline (PBS) prior to lymphocyte isolation by centrifugation at 20 °C in a gradient of Ficoll-Paque Plus at 400 g for 30–40 min. The band containing the lymphocytes was extracted and rinsed with PBS. Subsequently, cells were centrifuged at 1000 g for 10 min. This step was repeated three times. Finally, cells were fixed with 1% paraformaldehyde solution in 0.1 M phosphate buffer for 1 min. We tried different fixation conditions and ascertained that these parameters were the ones that worked best. Fixed lymphocytes could be stored at 4 °C for up to 1 week. For longer periods of time, they were stored at -80 °C in a cryoprotectant solution (Dimethyl-sulfoxide 10%; Fetal bovine serum 10% and RMPi-1640).

A total of 11 naïve male Long Evans (Charlers River) have been used in this analysis. All the procedures were conducted under a protocol approved by the Committee on Animal Care and Supply, Animal Research Ethics Board of the University of Saskatchewan (Canada). To obtain peripheral lymphocytes from rats, blood was drawn either by heart puncture (up to 3 ml in total, when animals were sacrificed for other research purposes) or directly from the tail vein (up to 1 ml). Blood was collected in 5 ml Eppendorf tubes containing blood and ADC solution in a 1:7 proportion. Centrifugation of samples, extraction and fixation of lymphocytes was carried out as explained above.

For SERT immunolabeling, samples were re-suspended in PBS and brought to room temperature, followed by centrifugation at 100 g for 10 min. To block unspecific staining, we performed a pre-incubation step for 10 min with 3% human IgG, or 3% rat IgG (I2511 and I4131, respectively, Sigma, St Louis, Missouri), and 1% bovine serum albumin (BSA, 3059-100Gr, Sigma, St Louis, Missouri) in PBS. For labeling of SERT, we used a polyclonal primary antibody raised in rabbit that specifically recognizes epitopes of both human and rat SERT [anti-serotonin transporter: AB10514P (Millipore, Billerica, Massachusetts)], and proceeded to incubate the lymphocytes with the primary antibody diluted 1:250 in 1% BSA in PBS at 4 °C overnight. After incubation with primary antibody, the samples were rinsed with PBS by centrifuging at 100 g for 10 min (repeated 3 times). After rinsing samples were then incubated for 1 h at room temperature with a 1:200 solution of the secondary antibody [Alexa Fluor 568, goat anti-rabbit: (A-11008, Molecular Probes, Eugene, Oregon)] in 1% BSA in PBS. This step is light sensitive, and therefore was carried out in the dark. After 3× rinsing with PBS (by centrifuging 3 times, 10 min each, at 100 g) the samples were re-suspended in 100-200 μl PBS. Sets of 20 μl from the samples were deposited on Super-Frost Plus slides (Menzel-Glaser, Braunschweig, Germany), coverslipped, and stored in the freezer at -20 °C.

### Immunocytochemistry of SERT in blood smears

Smears were prepared from blood collected by a quick prick on the tip of the forefinger in 6 human control subjects and a prick of the lateral tail vein in 19 male Long Evans untreated rats. Thin smears were spread on Super-Frost Plus slides, dried at room temperature for at least 1 hr., and then stored at 80 °C until used for immunocytochemistry.

We tested different fixation conditions: formalin 10% incubated for a minute, methanol incubated for 2 min, precooled acetone incubated at -20C for 10 min and at 4C for 2 h; the quality of the immunolabeling was not as good (see Discussion). We obtained the best results if the smears were fixed with 0.5 ml of 1% PFA for 5 min at room temperature. The fixation step was performed on dry slides, which were then washed and dried before being stored at -80 °C. We also examined the results if fixation was done in unfixed frozen slides brought back at room temperature and allowed to dry. There were no differences in the data obtained with either of these protocols.

Immunocytochemistry was performed directly on the smears using a protocol that was slightly modified from the one used on isolated lymphocytes. Smears include a whole blood sample: red blood cells, platelets and white blood cells, this causes more unspecific background with the immunolabelling. To avoid this, we have performed a pre-incubation step for 1 h at room temperature with 10% human IgG, or rat IgG diluted in 1% BSA and PBS. In the same way as the described protocol for isolated lymphocytes, samples were incubated overnight at 4C with primary antibody (same antibody as indicated above). After incubating, smears were rinsed with PBS for 10 min (3 times). The samples are subsequently incubated with fluorescent secondary antibody for 2 h at room temperature (same secondary antibody specified previously). We rinsed the excess of antibody by rinsing the sample with PBS 3 times 10 min each. To facilitate the identification of the lymphocytes, we used a nuclear marker [Hoechst 33,258 (Invitrogen, Molecular Probes, Carlsbad, California), applied at 2 μg/ml for 15 min at room temperature]. After nuclear labeling, we rinsed the smears with PBS for 10 min, let them dry, and coverslipped them. The slides were then stored in the freezer at -20 °C.

### Imaging processing and quantification

We analyzed SERT membrane protein clustering in isolated lymphocytes and in smears from both human and rat samples We obtained pictures at 100× magnification from 100 individual lymphocytes per sample using a fluorescence microscope. The pictures were subsequently analyzed using ImageJ software (1.48v, NIH, USA). We need to set appropriate conditions to accurately analyse the size of membrane protein clusters in both smears and isolated lymphocytes. ImageJ performs the measurements in pixels, so the first step must be the calibration of the program to get real values in our measurements (i.e. number and size of the SERT clusters). This process implies introduce a known distance to the program to establish equivalence in pixels to the distance given in μm. In a second step, we must adjust the background conditions in ImageJ to facilitate the analysis of the clusters. We will go to menu **Process → Substract** background and in **Rolling Ball Radius** we will indicate the size of the smallest particle which is not background (approximately 5–10 to isolated lymphocytes, and 1–3 in smears). When working with smears, an additional step is needed, **Remove Outliers: Radius 1 and Threshold 50,** in this way we can remove small dots that might interfere in the analysis and give us an overestimation of the number of clusters or an underestimation of the size. Following this step, a binary image is created where the clusters are easily quantified. However, if two clusters appear too close, the software could interpret as only one, and so provide an underestimation of the number of clusters and an overestimation of their size. To avoid this effect, we will add one more editing in menu **Process → Binary → Watershed.** In a third step, we will proceed to measure and quantify the clusters. By **Analyze → Measure**, the software gives us the measurement area, and by **Analyze → Analyze Particles**, the program provides a number and measure of the clusters. It is necessary indicate the minimum size of particle that the program will analyze to avoid a mistaken measurement. We have set that **0.05-Infinite** provides an accurate measure of the clusters. As an additional setting to gather an accurate measure will be indicate analyze **Pixel Units**.

Importantly, once we established a set of useable conditions in ImageJ, we kept them constant to make the results comparable across conditions. From the binary image of each lymphocyte we obtain two sets of measurements: the number of SERT expressing clusters, the size of those clusters. We average the measurements from each lymphocyte within each sample, and used to compare results between analysis done in smears and in isolated lymphocytes.

### Statistics

For the statistical analyses, we used SPSS 20 (IL, Chicago, USA). We compared the number and size of SERT protein clusters in lymphocytes analyzed from each protocol using two-tailed Student’s T-test in both human and rodent samples. The criterion for statistical significance was set at *p* < 0.05.

## Results

**Fig. 1:**
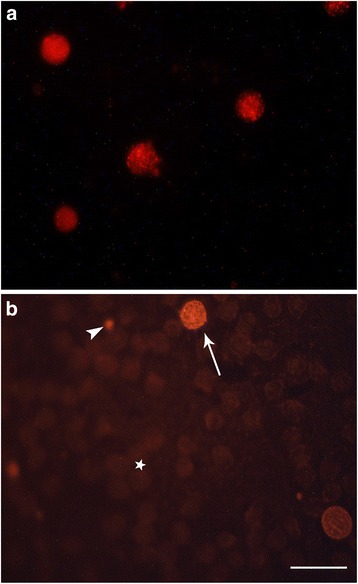
Images of lymphocytes immunostained for SERT in preparations of isolated lymphocytes extracted from whole rat blood samples (**a**), and in rat blood smears (**b**). Note than in rat blood smears lymphocytes can be easily identified (arrow), and are interspersed with numerous SERT immune-negative red blood cells (asterisk), and platelets that also show inmmunoreactivity for SERT (arrowhead). Calibration Bar: 10 μm

We compared two different methods to analyze SERT protein clustering in lymphocytes obtained from human and rat peripheral blood samples. Panel 1 shows low magnification images of isolated rat lymphocytes (Fig. 1a), and rat blood smears (Fig. 1b) that were immunostained for SERT. Although logically the number of lymphocytes per microscope field is higher in images from isolated lymphocyte preparations, the presence of lymphocytes is also easily seen in blood smears (arrow in Fig. 1b), where they are shown interspersed with red blood cells (asterisk), and platelets (arrowhead). Platelets also show SERT immunoreactivity, but are easily distinguished from SERT immunoreactive lymphocytes.

**Fig. 2:**
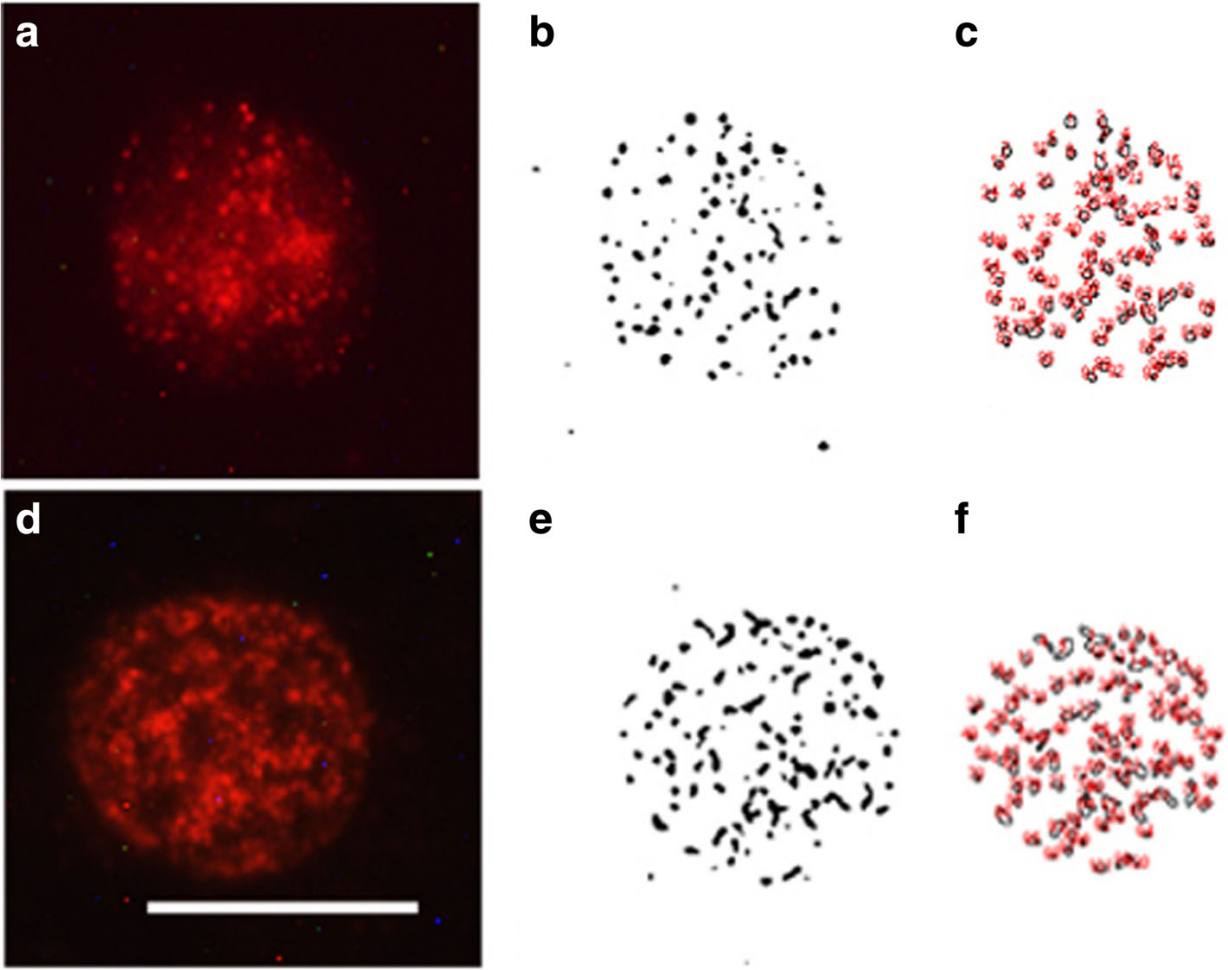
Example of analysis of SERT protein clusters in one rat lymphocyte isolated from whole blood samples (**a-c**), and one lymphocyte from rat blood smears (**d-f**). Figs. **a** and **c** show the microscope image of SERT immunostaining, while Figs. **b** and **e** represent the binary image as obtained in Image J, and Figs. **c** and **f** show the actual counts of the number and size of cluster as performed by the image analysis system. Calibration Bar: 10 μm

**Fig. 3:**
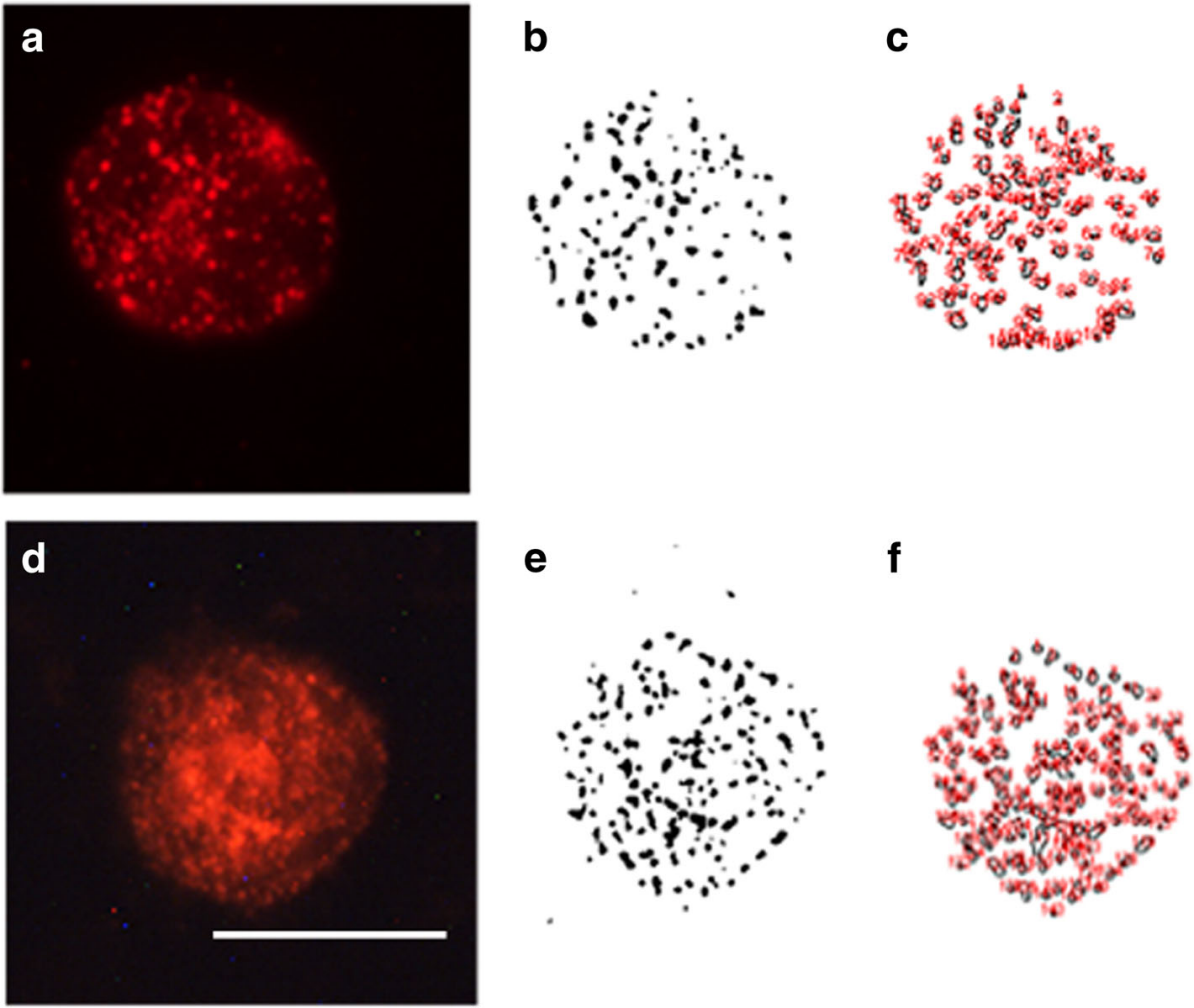
Example of analysis of SERT protein clusters in one human lymphocyte isolated from whole blood samples (**a-c**), and one lymphocyte in blood smears from human control subjects (**d-f**). Figs **a** and **c** show the microscope image of SERT immunostaining, while Figs. **b** and **e** represent the binary image as obtained in Image J, and Figs. **c** and **f** show the actual counts of the number and size of cluster as performed by the image analysis system. Calibration Bar: 10 μm

Once we determined the feasibility of SERT immunolabeling in lymphocytes within blood smears, we proceeded to carry out a quantitative study of SERT clusters on the plasma membrane of lymphocytes, in both isolated lymphocyte preparations and in blood smears obtained from rats and humans. In Fig. 2, we show an example of SERT clustering quantification in one isolated lymphocyte from a rat (A), and after converting the image to a binary colour and subtraction of background (B), the image was automatically analyzed by the Image J software to quantify the number of clusters per lymphocyte and average size of SERT clusters (C). The same composition of the analysis of one lymphocyte from a rat blood smear is shown in Fig. 2 d-e. In a similar way, Fig. 3 portrays images of the analysis of SERT clustering in isolated human lymphocytes (A-C), and in human blood smears (D-E).

**Fig. 4:**
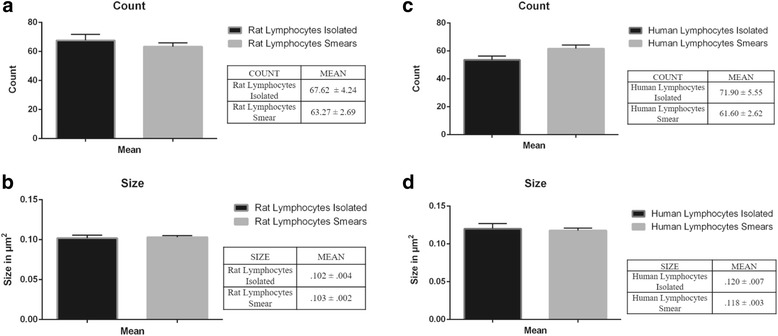
Histograms of the counts of SERT clusters number and size in isolated lymphocytes and lymphocytes in blood smears from rat and human control samples. Note that after protocol optimization the results obtained in the analysis of SERT clusters and size are quite similar

Figure 4 shows the quantified number and size of clusters in both rat (A-B) and human lymphocytes (C-D) from isolated lymphocyte preparations and blood smears. Our statistical analyses of these data revealed no significant differences in the number of clusters and their size when we compare the two protocols in rats [Number: t (28, .910); *p* = .371; Size: t (28, −.278); *p* = .783] or humans [Number: t (14, 1.366); *p* = .193; Size: t (14, .253); *p* = .804].

**Fig. 5:**
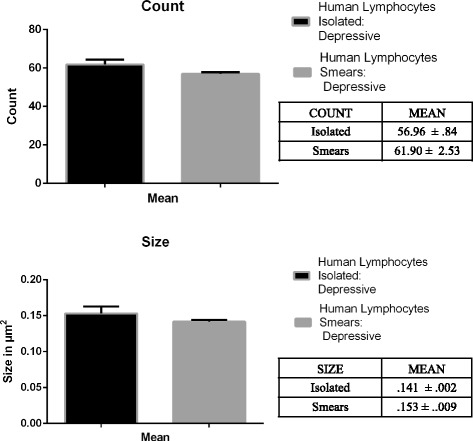
Histograms of the counts of SER clusters number and size in isolated lymphocytes and lymphocytes in blood smears from naïve depression patients. Note that after protocol optimization the results obtained in the analysis of SERT clusters and size are quite similar

Figure 5 shows the quantitative comparison of number and size of SERT clusters in isolated lymphocytes or blood smears from depression patients. There are no significant differences in the number and size of clusters when comparing the two protocols [Number: t (14, 1.032); *p* = .324; Size: t (14, .631); *p* = .541].

**Fig. 6:**
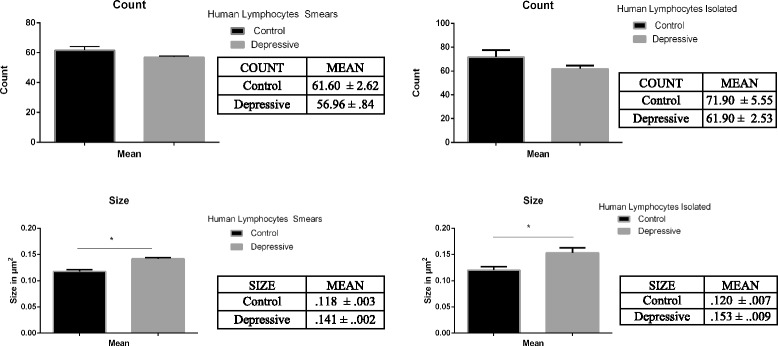
Histograms of the counts of SER clusters number and size in isolated lymphocytes and lymphocytes in blood smears from human control subjects and depression patients. Note that after protocol optimization the results obtained in the analysis of SERT clusters and size are quite similar for both protocols, indicating in both cases a lack of significant changes in the number of SERT clusters, and an increase in SERT clusters size in naïve depression patients

Figure 6 shows the quantitative analysis of SERT clustering in lymphocytes from human control subjects and depression patients, when using isolated lymphocytes or blood smears. Statistical analysis reveals no alterations in SERT numbers for both protocols; and an increase in SERT clusters which is similarly shown in both protocols [SERT Number blood smears: t (14, 1.687); *p* = .143; SERT number isolated lymphocytes: t (14, 1.639); *p* = .119; SERT size blood smears: t (14, −4.858); *p* = .002; SERT size isolated lymphocytes: t (14, −2.810); *p* = .012].

## Discussion

The identification of biomarkers to support a proper diagnosis, prognosis, and evaluation of therapeutic efficacy of antidepressant response is of the utmost importance [8]. In this experiment, we evaluated the feasibility of analysing membrane protein clusters in lymphocytes from blood smears, as this would provide a much easier and cheaper way to obtain appropriate samples in a clinical setting and it would also facilitate the study of lymphocytes in a research environment. Our results indicate that when the smears protocol is optimized, the data obtained from this approach are comparable to the data obtained when using isolated lymphocytes.

Our previous studies have been performed with isolated lymphocytes from peripheral blood samples, and we had adjusted the fixation, immunocytochemistry protocols, and image analysis settings to provide an accurate and replicable measurement of the number and size of membrane protein clusters (i.e., SERT and serotonin 2A receptors) in lymphocytes [5–7]. In this experiment, we developed a new protocol for analysing membrane protein clustering in lymphocytes in blood smears and optimized it to make it comparable to the analysis of protein clustering in isolated lymphocytes. We found that the major challenges associated with making the protocols comparable were related primarily to fixation and immunocytochemistry. For the fixation and immunolabeling of isolated lymphocytes, we use centrifugation (to obtain a pellet) and shaking the samples in a vortex (to re-suspend the samples) in every step, whereas we found that in the case of blood smears, we could conduct the fixation and immunolabeling in one spread layer of cells dried onto a coverslip. In these conditions, we determined after trying different fixation protocols, with different concentrations of PFA, at different temperatures, and for different periods of times, that 1% PFA for 5 min provides the most accurate results for immunolabeling of SERT clusters. This time is longer than the time required for fixation of isolated lymphocytes (i.e., which is 1 min), but it ensures that the quality of the immunolabeling in smears is similar to immunolabeling obtained in isolated lymphocytes (see Figs. 2 and 3). Our preliminary experiments also asserted that similar fixation characteristics are valid when using other anti-SERT antibodies or when labeling other membrane receptors and transporters (Romay-Tallon, personal communication).

Immunolabeling of SERT in smears also requires an increase in incubation time with at almost each step of the process. This is primarily because with smears the cells are adhered to the slide and therefore the labeling of clusters in the cell membranes requires a longer period of exposure to antibodies than what would normally be necessary for immunolabeling re-suspended cells, as it is the case for isolated lymphocytes. We have also discovered that the temperature at which incubation with the primary and secondary antibodies occurs is vital both for smears and isolated lymphocytes, as other temperature settings during these steps (e.g., incubation at room temperature) results in an increase in size and decrease in number of SERT clusters.

Finally, it is important to define a set of conditions for the image analysis in ImageJ software (e.g. background clearing conditions, define the minimum size of a particle that would be quantified) that offer a realistic representation of the lymphocytes which provides a more accurate analysis of the membrane protein clustering. It is crucial to keep these settings for all subsequent analyses to compare the results across all the analyzed groups.

When complying with all these conditions, the analysis of SERT protein clustering in cell membranes from lymphocytes in blood smears is as accurate as the analysis done in isolated lymphocytes, and provides similar results (Fig. 4).

Our previous studies focusing on the pattern of SERT labeling in isolated lymphocytes from control subjects and depression patients allowed us to differentiate two subpopulations of drug naïve depression patients that respond differently to antidepressant drugs. More specifically, we identified a group called D-I patients, which had more SERT clusters per lymphocyte but smaller SERT cluster size than the second group called D-II patients [5]. The D-I group showed very little response to antidepressant drugs after 8 weeks of treatment, but the D-II group achieved a full remission of symptoms after 8 weeks of treatment. We concluded from these observations that the pattern of SERT protein clustering in lymphocytes could be a putative biomarker of therapeutic responsivity for antidepressant medication [5–7]. Although these findings are important, one of the difficulties inherent in translating these data to a clinical setting relates to the fact that most naïve depression patients are diagnosed, assessed, and get their prescriptions in a family clinic setting, where appropriate conditions for routinely drawing blood samples for experimental evaluation of this biomarker may not be present. To circumvent this problem, we focussed on developing an alternative protocol by adapting our methodology to work with blood smears that are easily obtained and can be collected in an inexpensive way. In fact, the present results indicate that alterations in SERT clustering in blood smears between human control subjects and naïve depression patients are similar to those observed when using isolated lymphocytes, thereby validating the use of blood smears for quantitative analysis of membrane protein clustering in the context of further development of this technology as a biomarker of depression.

## Conclusions

The development of this protocol will allow us to quickly advance the validation of SERT membrane protein clustering in lymphocytes as a biomarker of therapeutic efficacy in major depression, and facilitate the implementation of this type of analysis in clinical settings.

## Abbreviation

SERT: Serotonin transporter
